# Identification of yellow vein clearing disease in lemons based on hyperspectral imaging and deep learning

**DOI:** 10.3389/fpls.2025.1554514

**Published:** 2025-06-16

**Authors:** Xunlan Li, Fangfang Peng, Zhaoxin Wei, Guohui Han

**Affiliations:** Research Institute of Pomology, Chongqing Academy of Agricultural Sciences, Chongqing, China

**Keywords:** hyperspectral imaging, disease identification, lemon, machine learning, deep learning

## Abstract

Hyperspectral imaging (HSI) technology has great potential for the efficient and accurate detection of plant diseases. To date, no studies have reported the identification of yellow vein clearing disease (YVCD) in lemon plants by using hyperspectral imaging. A major challenge in leveraging HSI for rapid disease diagnosis lies in efficiently processing high-dimensional data without compromising classification accuracy. In this study, hyperspectral feature extraction is optimized by introducing a novel hybrid 3D-2D-LcNet architecture combined with three-dimensional (3D) and two-dimensional (2D) convolutional layers—a methodological advancement over conventional single-mode CNNs. The competitive adaptive reweighted sampling (CARS) and successive projection algorithm (SPA) were utilized to reduce the dimensionality of hyperspectral images and select the feature wavelengths for YVCD diagnosis. The spectra and hyperspectral images retrieved through feature wavelength selection were separately employed for the modeling process by using machine learning algorithms and convolutional neural network algorithms (CNN). Machine learning algorithms (such as support vector machine and partial least squares discriminant analysis) and convolutional neural network algorithms (CNN) (including 3D-ShuffleNetV2, 2D-LcNet and 2D-ShuffleNetV2) were utilized for comparison analysis. The results showed that CNN-based models have achieved an accuracy ranging from 93.90% to 97.35%, significantly outperforming machine learning approaches (ranging from 68.83% to 93.52%). Notably, the hybrid 3D-2D-LcNet has achieved the highest accuracy of 97.35% (CARS) and 96.86% (SPA), while reducing computational costs compared to 3D-CNNs. These findings suggest that hybrid 3D-2D-LcNet effectively balances computational complexity with feature extraction efficacy and robustness when handling spectral data of different wavelengths. Overall, this study offers insights into the rapidly processing hyperspectral images, thus presenting a promising method.

## 1 Introduction

Lemon (C. *limon*), a widely cultivated popular fruit, faces a significant threat from the citrus yellow vein clearing virus (CYVCV), which has caused substantial losses to the lemon industry, particularly in China and other major production countries (Liu et al., 2020). This disease severely affects the health and yield of lemon trees, often leading to orchard destruction. The affected areas are gradually expanding in major production regions such as China, India, and Pakistan (Liu et al., 2020). Moreover, no effective pesticides have been found to combat this viral disease once the trees become infected. The primary method for controlling the yellow vein clearing disease (YVCD) in lemons involves identification, quarantine and eradication of diseased trees. Currently, the traditional manual survey method and polymerase chain reaction (PCR)-based molecular technique (Chen et al., 2016; Afloukou and Önelge, 2021) are the main methods for identifying YVCD in lemons. Current methods such as manual survey and PCR-based detection have their limitations in light of time and cost invested, and environmental concerns, which has reduced their practical applicability for large-scale early diagnosis (Martinelli et al., 2015). Furthermore, multiple diseases often co-occur on a single lemon tree, of which the leaves exhibit the complex symptoms of YVCD and other diseases, thus making it more difficult to identify YVCD. Rapidly and accurately identifying a disease from leaves exhibiting complex symptoms is essential for effective disease control, which provides valuable guidance for orchard field management. There is an urgent need to develop more effective methods for YVCD detection when it comes to supporting the sustainable development of the lemon industry.

In recent years, optical and imaging techniques have been widely applied to the rapid identification of plant diseases (Pereira et al., 2011; Calderón et al., 2013; Poblete et al., 2021; Vallejo-Pérez et al., 2021). Hyperspectral imaging (HSI) technology surpasses the traditional imaging and spectroscopy techniques owing to its superior spectral resolution and broader wavelength ranges. HSI can acquire both spatial and spectral information of the plant tissue (Thomas et al., 2018). These advantages allow HSI to capture spectral changes caused by subtle alterations in plant physiological and metabolic status, which is conducive to more accurate and earlier detection of plant diseases (Mishra et al., 2017). HSI has been proven effective in identifying citrus diseases. For example, Weng et al. (2018) had achieved an accuracy of 93% in detecting Huanglongbing using HSI. In laboratory settings, Abdulridha et al. (2019) achieved accuracies of 94%, 96%, and 100% in detecting asymptomatic, early, and late symptom stages of citrus canker, respectively. Additionally, Guo et al. (2015) had achieved an accuracy of over 97% in detecting citrus tristeza virus. Satisfactory identification results have also been obtained in detecting other crop diseases. For instance, Lu et al. (2018) reported 100% accuracy in detecting yellow leaf curl disease in tomato leaves, and Liu et al. (2024) achieved 99.38% accuracy in identifying apple mosaic virus. Therefore, HSI has the potential to be applied in developing effective and accurate techniques for identifying YVCD in lemons. Despite the advantages, HSI involves complex data processing and calibration, which must be addressed for field-level deployment.

Hyperspectral images encompass both spectral and spatial information. In previous studies, spectral information was applied in developing disease identification technology, with spatial information neglected. Traditional processing of hyperspectral images mainly involves spectral preprocessing, feature extraction and model construction. Machine learning algorithms are widely used and have achieved excellent performance in the field of classification (Guo et al., 2015; Nagasubramanian et al., 2018; Weng et al., 2018; Jiang et al., 2021; Chen et al., 2025). Partial least squares and support vector machines (SVM) are two commonly used methods, which have good performance in data fitting and classification tasks (Xuan et al., 2022; Siripatrawan and Makino, 2024). In recent years, convolutional neural networks (CNNs), which can automatically extract and learn the most significant features in an end-to-end manner, have been extensively used in disease diagnosis and hyperspectral image processing (Lu et al., 2021; Russel and Selvaraj, 2022; Kaya and Gürsoy, 2023). Hyperspectral images can be considered as three-dimensional hypercubes encompassing spectral and spatial information. Both two-dimensional and three-dimensional CNNs can extract and learn features of this three-dimensional cube data. A three-dimensional convolutional neural network (3D-CNN) can extract both spectral and spatial features simultaneously, whereas, a two-dimensional neural network (2D-CNN) only captures spatial features. As a result, the rich spectral information of hyperspectral images is often compressed and not completely extracted (Jia et al., 2023). Ortac and Ozcan (2021) compared the performance of one-dimensional, two-dimensional, and three-dimensional CNNs in hyperspectral image classification. They reported that the 3D-CNN effectively integrated spectral and spatial information, achieving a higher classification rate. Similarly, Nguyen et al. (2021) used hyperspectral imaging and deep learning for early detection of grapevine virus. They reported that 3D-CNNs were more effective than 2D-CNNs in extracting features from hyperspectral images. This trend of 3D-CNNs outperforming 1D-CNN and 2D-CNN was further validated by Zhu et al. (2023), who applied HSI and CNNs to identify slightly sprouted wheat kernels. The above results indicate that 3D-CNNs have demonstrated superior performance in HSI classification tasks, although their advantages may depend on dataset characteristics and model configuration. However, compared to 2D convolution, 3D convolution requires computing an additional depth channel in performing the convolution, which increases the number of parameters, FLOPS, and training time in modeling. Therefore, while 3D-CNNs have demonstrated better feature integration, their scalability and field application are limited by high computational cost. Hybrid CNNs combine the representational power of 3D convolutions with the computational efficiency of 2D convolutions, enabling better generalization with fewer parameters. In recent years, studies have shown that hybrid CNNs combining 3D and 2D convolution could further improve classification performance when hyperspectral images are employed. Roy et al. (2020) proposed a hybrid 3D and 2D convolution spectral CNN (HybridSN) for HSI classification, achieving satisfactory performance. Qi et al. (2023) proposed a deep learning architecture (PLB-2D-3D-A) combining 2D and 3D convolutions to identify potato late blight disease. With higher accuracy, this architecture outperformed the random forest and the deep learning-based methods 2D-CNN and 3D-CNN. Chen et al. (2022) constructed three CNNs (2D-CNN, 3D-CNN, and 2D–3D-merged CNN) for inspecting defects in green coffee beans, resulting in the 2D-3D-merged CNN based on both full band and PCA-3 bands achieving a higher accuracy.

Currently, there are no reports on the rapid detection of YVCD in lemons with the use of HSI technology. The primary objective of this study was to investigate how to efficiently process hyperspectral images and achieve accurate identification of YVCD. The specific objectives are: 1) to conduct YVCD identification by selecting characteristic wavelengths; 2) to construct models based on spectral information and traditional machine learning algorithms, and evaluate the performance of traditional hyperspectral image processing methods in identifying YVCD; 3) to construct models on the basis of CNNs and evaluate their performance in identifying YVCD; and 4) to perform a comprehensive comparison to select the most efficient and accurate method for YVCD identification.

The key outcomes of this study represent three significant advances. First, a HSI-based rapid detection method for YVCD identification was developed, filling the gap of lacking effective and accurate identification techniques for this disease. Meanwhile, the characteristic wavelengths were screened on the same dataset by using the CARS and SPA independently, which may support future development of low-cost, portable detection tools. Furthermore, a new 3D-2D-LcNet architecture has been proposed. This architecture can effectively combine spatial and spectral feature extraction, optimally balancing computational complexity and feature extraction efficiency in hyperspectral image processing, thus achieving high classification accuracy. These advancements not only are able to tackle the dimensional challenges in hyperspectral image processing but also help build a scalable technical foundation for plant disease monitoring.

## 2 Materials and methods

### 2.1 Hyperspectral image acquisition

**Figure 1:**
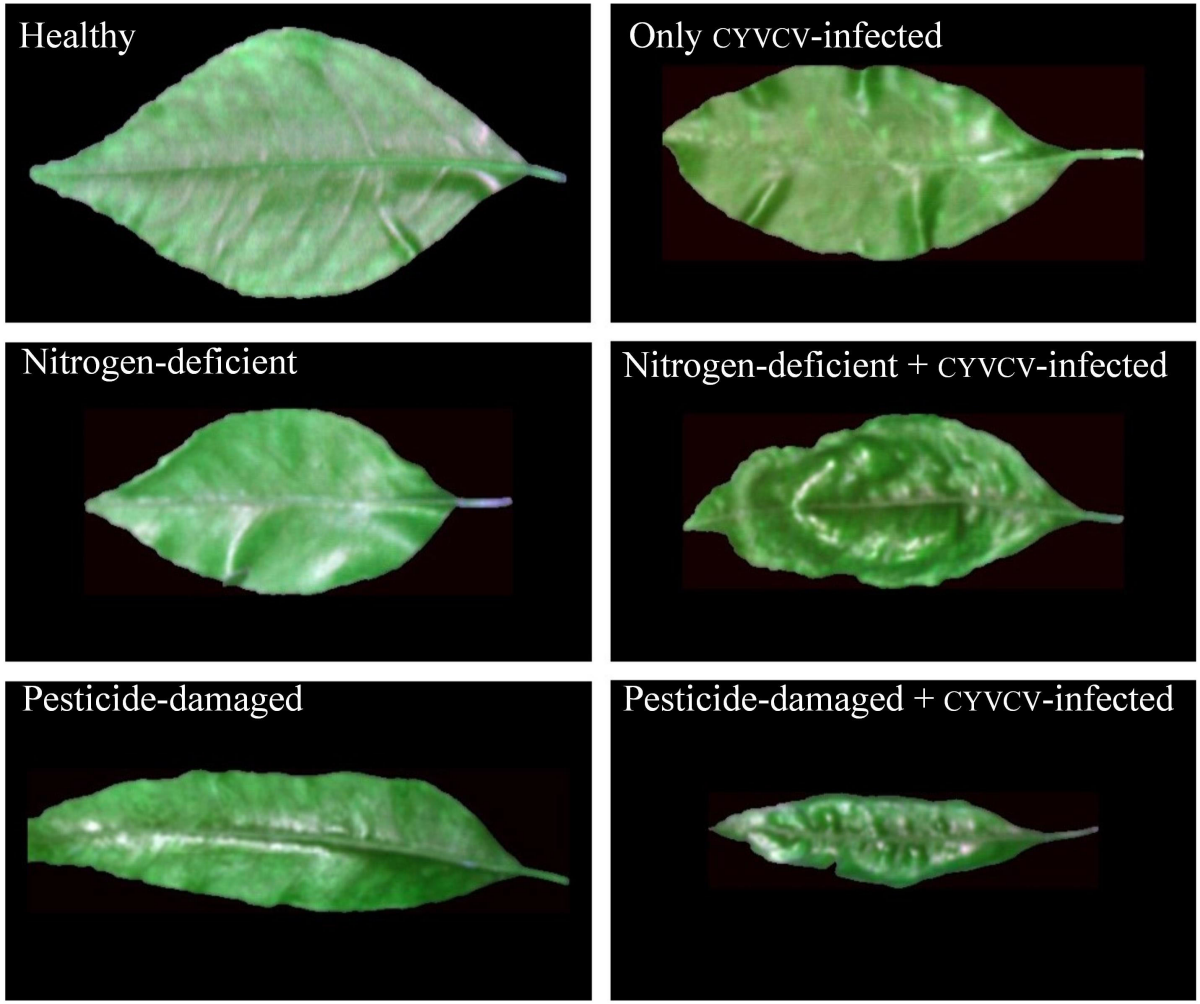
Samples of lemon leaves.

Totaling 522 4-month-old leaves were collected from lemon trees grown in a lemon orchard in Tongnan District, Chongqing City, China, in 2022. This orchard is a newly documented spread area for CYVCV, with only a few plants infected. Plant protection experts have identified the infected leaves and classified them into six groups on the basis of the presence, absence, or combination of disease symptoms (**Figure 1**): healthy (90 leaves, no symptoms of disease, nutrient deficiency, or pesticide damage), CYVCV-infected only (90 leaves, exhibiting specific symptoms of CYVCV with no signs of nutrient deficiency or pesticide damage), nitrogen-deficient (79 leaves, displaying nitrogen deficiency symptoms) without CYVCV infection or pesticide damage, nitrogen-deficient + CYVCV-infected (81 leaves, showing combined symptoms of nitrogen deficiency and CYVCV infection, including yellowing leaves with viral vein clearing or distortion), pesticide-damaged (90 leaves, With morphological signs of pesticide injury but no CYVCV infection or nutrient deficiency), and pesticide-damaged + CYVCV-infected (92 leaves, presenting mixed symptoms of pesticide damage and CYVCV infection). This orchard is a newly documented spread area for yellow vein disease, with only a few plants infected. All CYVCV-infected-free plants are located outside a range of 1000 m from the infected ones. The healthy, nitrogen-deficient only, and pesticide-damaged only groups were collected from the non-infected areas of the orchard. During sample categorization, the leaves of all diseased groups showed mild, early-stage, or indistinct symptoms.

**Figure 2:**
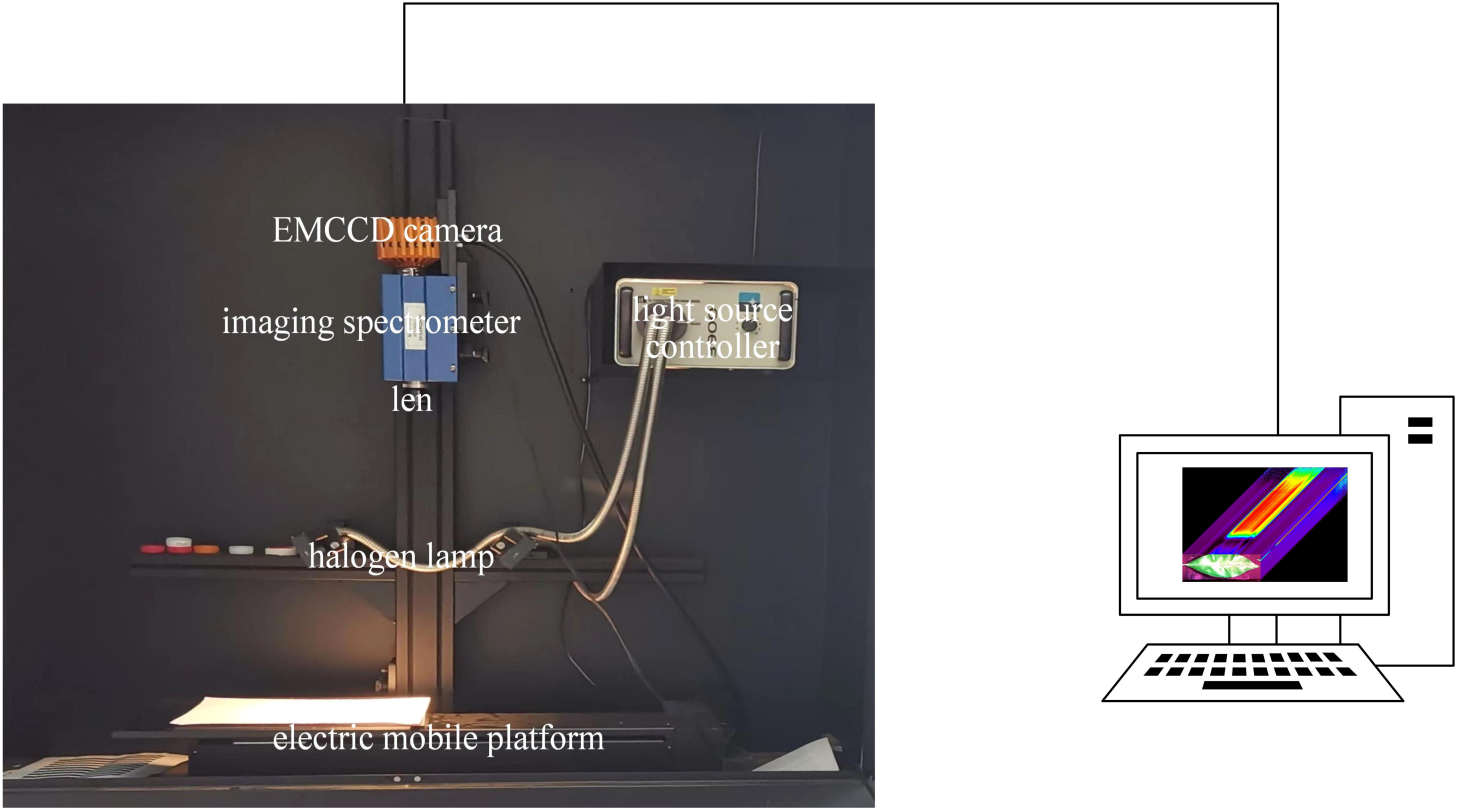
The hyperspectral image acquisition system.

We used clean water to wash the leaves, and subsequently acquired the hyperspectral images with the HSI acquisition system (**Figure 2**). The HSI acquisition system comprised a darkbox (a light-tight enclosure), a hyperspectral imaging spectrometer (ImSpector V10E, Spectral Imaging Oy Ltd, Finland), an electric moving platform and controller (SC30021A, Zolix, China), two halogen light sources (150 W/21 V halogen lamp, Illuminator Technologies, Inc., USA) and a laptop. The spectrometer operated over a spectral range of 305–1090 nm. The halogen lamps were positioned at a 45°angle relative to the sample, with a fixed lens distance of 45 cm. The motorized stage moved at a speed of 1.87 mm/s. Before image acquisition, the system was preheated for 20 minutes to ensure stability and all measurements were conducted at an ambient temperature of 25°C to maintain consistent environmental conditions.

To eliminate the effects of uneven illumination and dark current noise, the white reference hyperspectral image was obtained by scanning a standard white board with 99% reflectance and a dark reference hyperspectral image was obtained by scanning with the lens closed. The original hyperspectral image was corrected by using the following Equation 1:


(1) $$I_{c}=\frac{I_{raw}-I_{dark}}{I_{white}-I_{dark}}$$

Where *I_raw_* represents the raw hyperspectral image, *I_dark_* represents the dark reference image, *I_white_* represents the white reference hyperspectral image and *I_c_* represents the corrected hyperspectral image.

**Figure 3:**
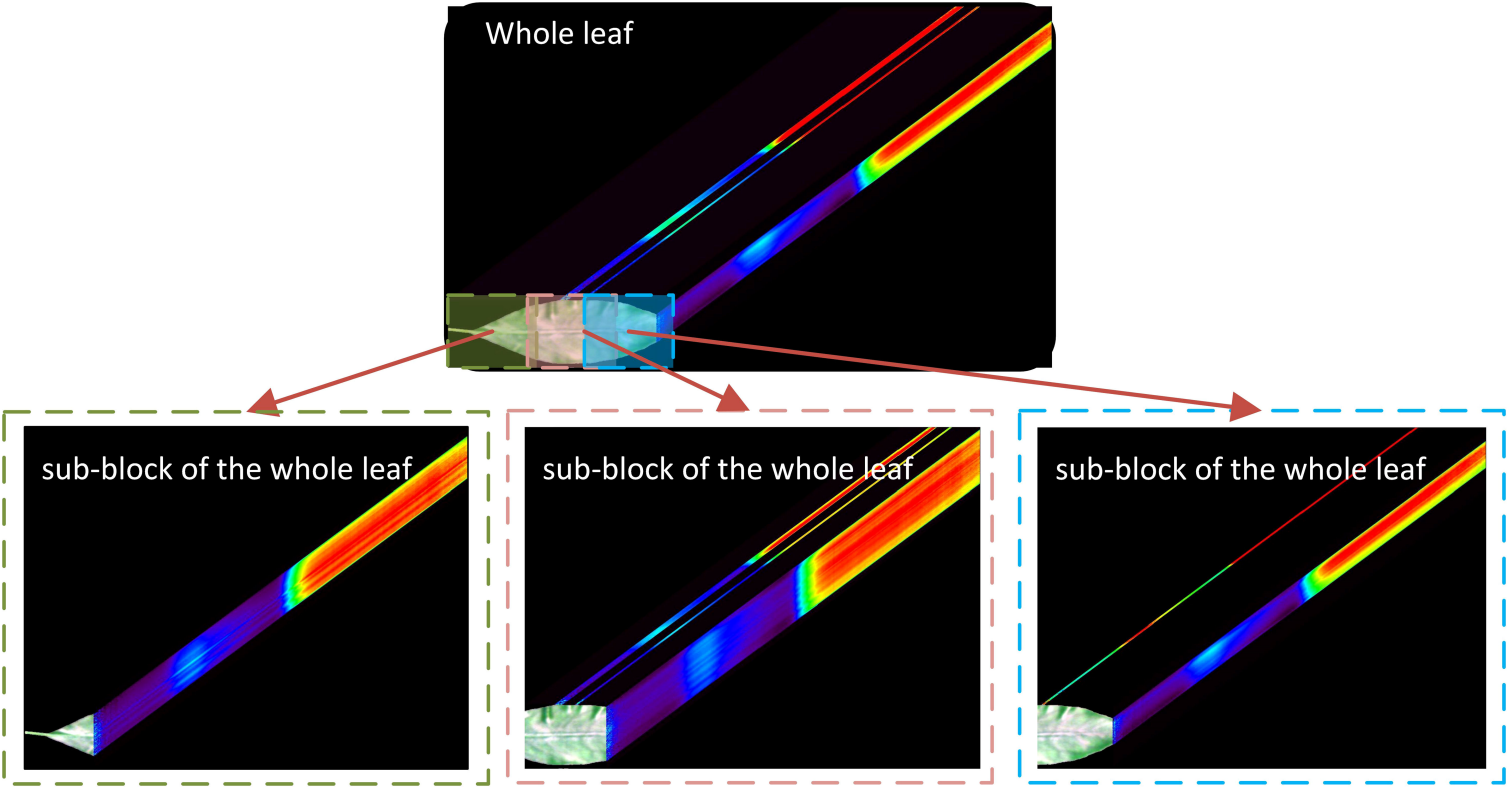
Hyperspectral images for machine learning and deep learning modeling.

Hyperspectral images datasets were randomly divided into training, validation, and testing sets in a 60:20:20 ratio. The high cost of obtaining hyperspectral images had limited the data set available for this study, which is insufficient for deep learning. To address this limitation, a whole leaf was divided into 2–4 sub-blocks after the initial division into sample sets by referring to the methods of Park et al. (2018) and Nagasubramanian et al. (2019). The segmented whole leaves and their corresponding sub-blocks hyperspectral images were subsequently utilized for subsequent data analysis. The detailed segmentation method is illustrated in **Figure 3**, where the overlap rate between every two sub-blocks is less than 30%. To avoid data leakage, the segmentation was performed after the datasets had been divided into training, validation, and test sets.

To ensure robustness and reduce variability in terms of CNNs model performance, we implemented 5-time repeated random splitting on the combined training and validation set (80% total data). In each repetition, 60% of the total data was randomly selected as training set with preserved class distribution, while 20% of total data served for validation. The independent testing set (20%) remained strictly isolated for final evaluation. Dunn’s test with Bonferroni correction was used to analyze the significant differences.

### 2.2 Traditional processing of hyperspectral images

#### 2.2.1 ROI segmentation and spectra extraction

At 800 nm, the spectral reflectance of the background and that of the leaf exhibited significant differences, which represents a characteristic facilitating the accurate separation of leaves from the background. Specifically, The grayscale image obtained at 800 nm was processed by using Otsu’s method (Yin et al., 2022). With the optimal segmentation threshold automatically determined, the grayscale images were partitioned into two classes: leaves (foreground) and background. This process generated a binary mask where pixel values of zero and one respectively denote the background and leaves. Then mask was applied to the full-band hyperspectral data cube, retaining only the spectral information of the leaf region while excluding background noise, thus achieving precise segmentation of leaves from the background. Finally, the spectral data of all pixels within the ROI of the leaf were averaged, and the mean spectrum was used for subsequent feature wavelength extraction and modeling.

#### 2.2.2 Characteristic wavelength extraction and modeling

To eliminate noise at the extremes of the spectrum and facilitate subsequent analysis, we used spectral bands ranging from 400 nm to 1000 nm, which encompassed 761 wavelengths. Standard normal variate (SNV) (Barnes et al., 1989) was used to mitigate the effects of diffuse reflection, which is induced by uneven particle distributions, varying particle sizes, and differences in path length. This method was chosen over others (e.g., multiplicative scatter correction, MSC) due to its superior performance in preliminary tests. Given the high correlation among adjacent spectral bands, the Successive Projections Algorithm (SPA) (Ye et al., 2008) and Competitive Adaptive Reweighted Sampling (CARS) (Li et al., 2009) were applied for characteristic wavelengths extraction. This approach aimed to reduce redundancy and interference, thus alleviating the computational load on the hardware. Support Vector Machine (SVM) (Boser et al., 1992) and Partial Least Squares Discriminant Analysis (PLS-DA) are well -known traditional supervised learning algorithms. In this study, both SVM and PLS-DA were employed for YVCD identification. During the modeling process, the grid search algorithm was carried out to optimize the kernel function parameters for SVM and the number of principal components for PLS-DA.

### 2.3 Processing of hyperspectral images via CNNs

#### 2.3.1 Dataset preparation

To standardize input dimensions and preserve central features, zero-value centered padding was applied to resize the ROIs to 112×112 pixels, as this size balances spatial resolution with GPU memory constraints. Subsequently, data augmentation was performed by using flip, rotation, and mirror techniques. Finally, the number of samples per type expanded to approximately 2000. The quality of the dataset significantly affects the training. Directly utilizing high-dimensional hyperspectral data for modeling not only strains computational resources but also degrades prediction accuracy due to the curse of dimensionality and redundant spectral features (Ram et al., 2024). Although CNNs can automatically extract features, it is essential to reduce the dimensionality of hyperspectral images to lower the training cost and enhance the prediction accuracy, especially when high spatial resolution or large spatial dimensions are used. In this study, hyperspectral images composed of extracted feature wavelengths were used for subsequent deep learning processes.

#### 2.3.2 CNN framework

**Figure 4:**
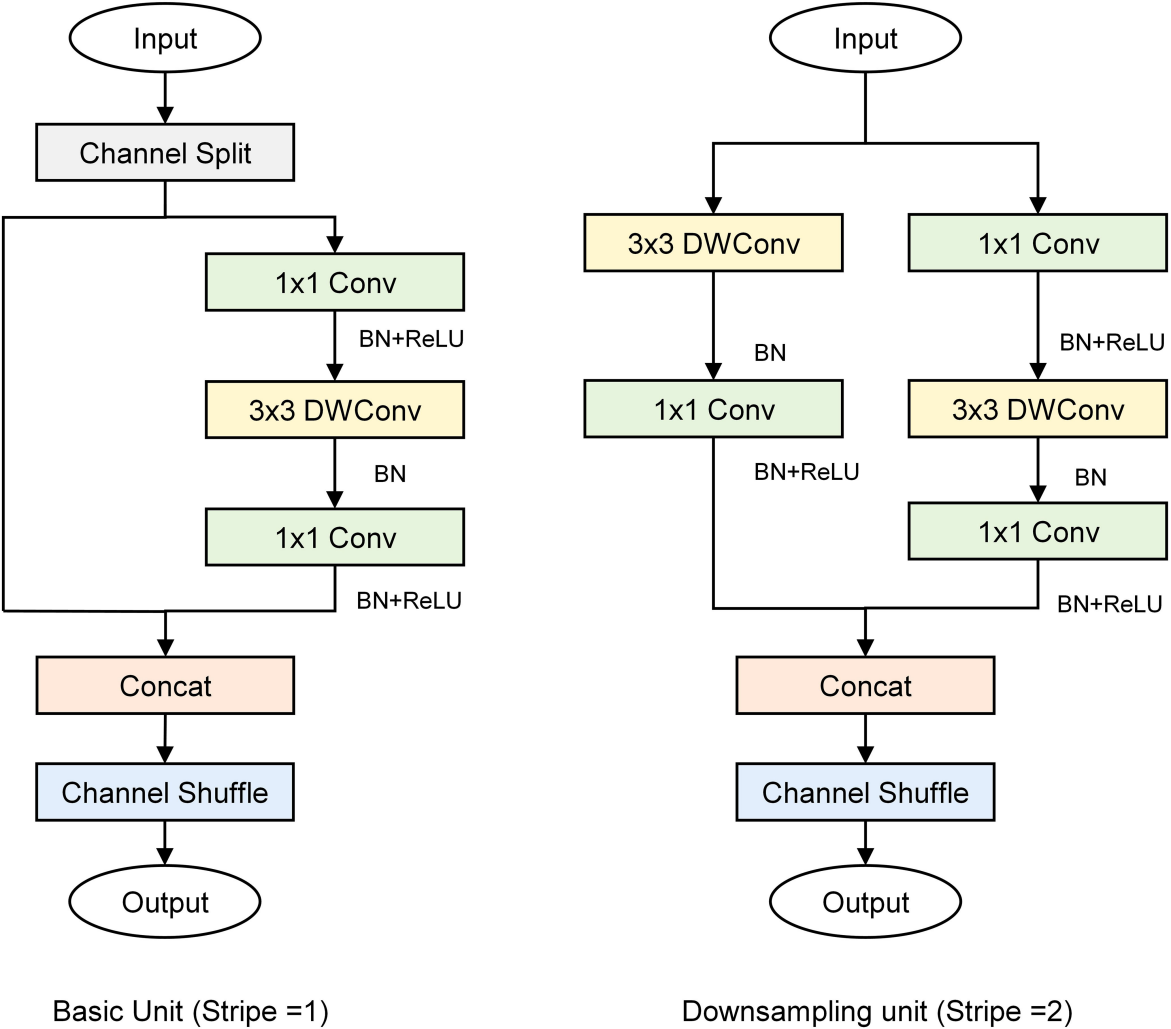
Building blocks of ShuffleNetV2.

ShuffleNetV2 (Ma et al., 2018) is a lightweight CNN that adopts grouped convolutions grouped convolution, depthwise separable convolution, and channel shuffle techniques to guarantee both its speed and accuracy. In ShuffleNetV2, the basic and down sampling units are the primary building blocks of the network (**Figure 4**). The basic unit introduces channel splitting, pointwise convolution, deep convolution, and channel shuffling to effectively balance the number of channels and spatial information processing, and enable the network to maintain high accuracy with low computational complexity. The down sampling unit splits the input into two parts, effectively reducing the feature map size while increasing the number of channels, thereby enhancing feature extraction.

This study presents 3D-2D-LcNet, a lightweight hybrid CNN architecture. Unlike conventional hybrid CNNs that employ standard 2D convolution blocks, our design integrates optimized ShuffleNetV2 basic units and downsampling units in the post-3D processing stage. Compared with the original ShuffleNetV2 architecture, these units are adapted by reducing layer repetitions and lowering channel dimensions to strike a balance between computational efficiency and feature extraction capability. Specifically, the channel shuffle mechanism in ShuffleNetV2 basic units enables cross-group feature channel mixing to enhance inter-channel information interaction, while its downsampling units minimize information loss during spatial dimension reduction. These improvements maintain the 3D to 2D sequential processing pipeline consistent with traditional models, and enable direct processing of large-scale hyperspectral images (112×112 pixels), effectively addressing the application limitation of conventional models that can only handle small-sized inputs (e.g., ≤15×15 pixels). The network processes input hyperspectral cubes (112×112×N, where N=15 or 30 spectral bands) through two initial 3×3×3 3D convolutional layers, with each followed by batch normalization and ReLU activation function for effective feature extraction and training stabilization. Subsequently, a 3D adaptive average pooling layer is employed to reduce the spectral dimension from 8 (or 4, depending on the preceding convolutional stage) to 2. The output is then reshaped into a 28×28×64 tensor, enabling a seamless transition to 2D convolutional processing for spatial feature extraction. The reshaped feature maps contain an excessive number of channels. Modified ShuffleNetV2 units are introduced for these reshaped features: (1) a downsampling unit (stride=2) to shrink spatial resolution to 14×14 and enlarge the channels to 128; (2) a basic unit (stride=1) to keep the 14×14×128 resolution through channel shuffling and pointwise convolutions; (3) a second downsampling unit (stride=2) to further decrease resolution to 7×7 and raise the channels to 256; and (4) a final basic unit (stride=1) to retain the 7×7×256 features. Finally, the network concludes with a 1×1 convolution expanding channels to 512, global average pooling, dropout (p=0.2) for regularization, and a softmax classifier, enabling efficient hyperspectral image analysis through this optimized 3D-2D hybrid architecture.

**Figure 5:**
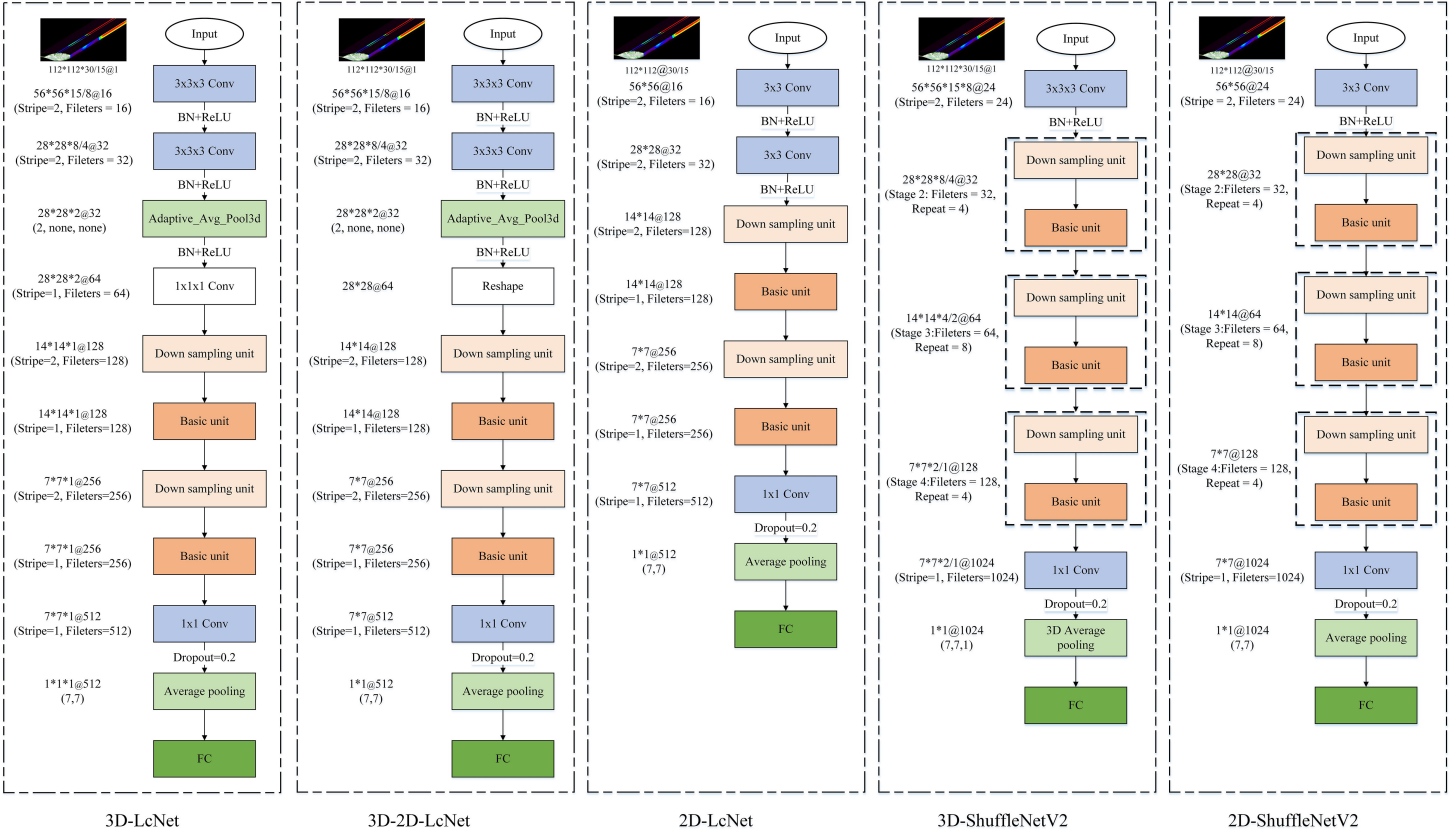
Architecture of CNNs.

To evaluate the performance of 3D-2D-LcNet in processing hyperspectral images for identifying YVCD, a comparative analysis was performed on the results generated by 3D-2D-LcNet, 3D-LcNet, 3D-ShuffleNetV2, 2D-LcNet, and 2D-ShuffleNetV2. Here LcNet refers to a lightweight convolutional network designed for efficient spatial-spectral feature fusion. For both 3D-ShuffleNetV2 and 2D-ShuffleNetV2, the width_mult was set to 0.25. Specifically, the output channels of the first and last convolutions were configured as 24 and 1024, respectively. The output channels of Stage 2, Stage 3, and Stage 4 were set to 32, 64, and 128, with repetition counts of 4, 8, and 4, respectively. The detailed architectures of the CNNs are shown in the **Figure 5**.

### 2.4 Experimental environment and evaluation

MATLAB R2020a was utilized for ROI segmentation, spectral extraction, and feature wavelength extraction. The training of the CNN models was carried out in the Anaconda environment via Python 3.9.13, PyTorch 1.11, and CUDA 11.3. The computer configuration comprised an ubuntu 20.04 system equipped with a 16-core Intel(R) Xeon(R) Gold 6430 CPU, an NVIDIA RTX 4090 GPU (24 GB VRAM), 120 GB RAM, and 1 TB storage.

In the CNN model training, the StepLR learning rate decay strategy was implemented to adjust the learning rate in order to enhance convergence and performance. The initial learning rate was set to 0.001, decaying to 50% of its previous value every 20 epochs. The total number of epochs was set to 200. The cross-entropy loss function was used. The optimizer used was the adaptive moment estimation (Adam) optimizer with its default parameters. The batch size was set to 64.

The confusion matrix along with precision, recall, F1-score, accuracy, and Matthews Correlation Coefficient (MCC) were used to evaluate the model performance. The calculation equations are presented in the following Equations 2-6:


(2) $$\text{Accuracy}=\frac{\text{TP}+\text{TN}}{\text{TP}+\text{FN}+\text{FP}+\text{TN}}$$

(3) $$Precision=\frac{TP}{TP+FP}$$

(4) $$Recall=\frac{TP}{TP+FN}$$

(5) $$F1-Score=\frac{2*Precision*Recall}{Precision+Recall}$$

(6) $$MCC=\frac{TP*TN-FP*FN}{\sqrt{\left(TP+FP\right)*\left(TP+FN\right)*\left(TN+FP\right)*\left(TN+FN\right)}}$$

Where *TP, TN, FP*, and *FN* respectively denote true positive, true negative, false positive and false negative.

To assess practical deployability, we quantified model efficiency using PyTorch’s built-in utilities: parameter count was obtained by *torchinfo.summary()*, FLOPS were calculated with the flops-counter package for a typical input size of 112×112×bands (bands =30 or 15), training time was logged by using NVIDIA CUDA events on an RTX 4090 GPU, and testing time represented the total inference duration for all independent samples in the test set (3,304 samples). Units were reported as millions of parameters (M), giga-floating-point operations (G-FLOPS), seconds (s) for training and testing.

**Figure 6:**
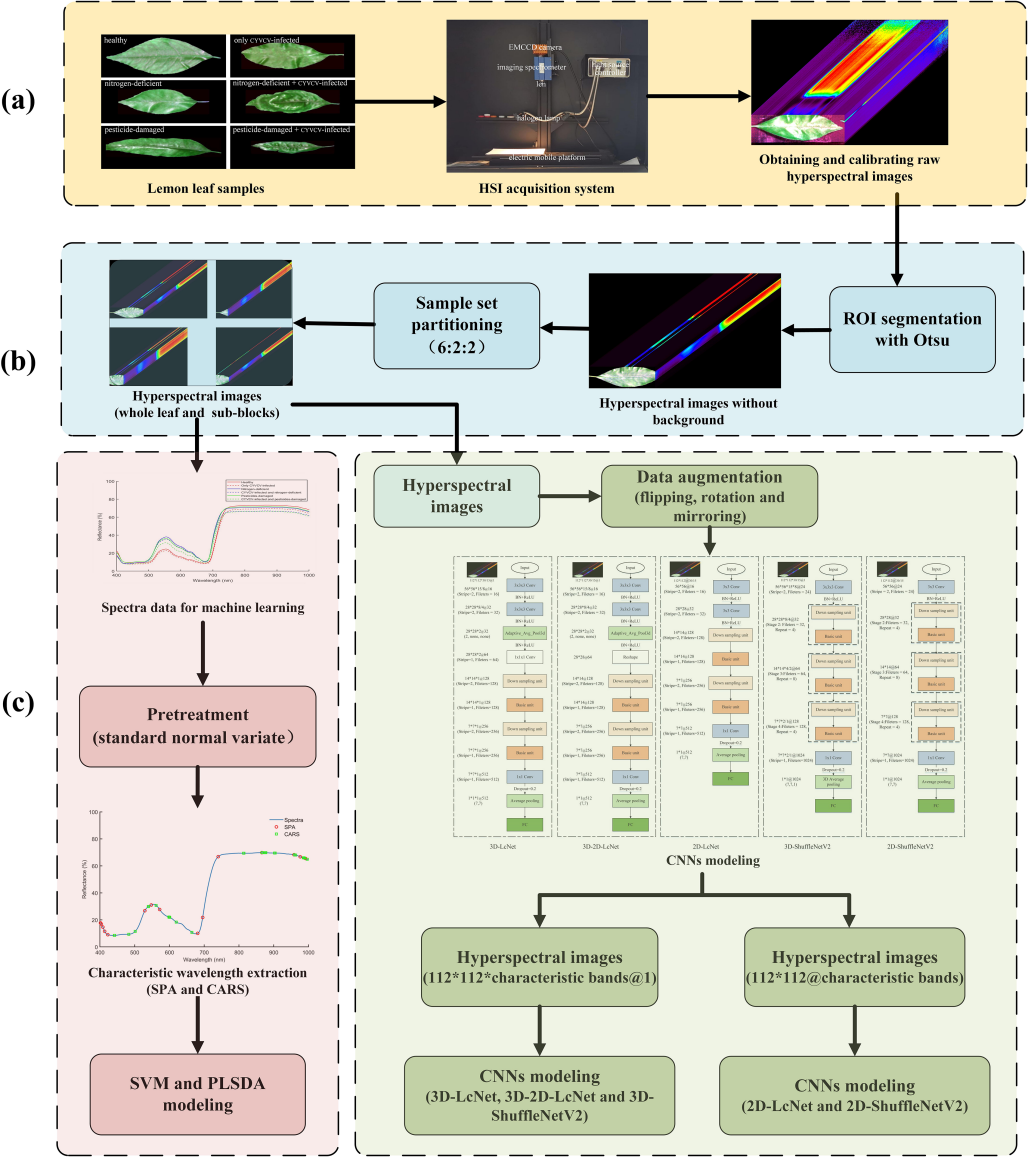
Research workflow. **(a)** Hyperspectral image acquisition; **(b)** ROI segmentation and spectra extraction; **(c)** characteristic wavelength extraction and modeling; **(d)** processing of hyperspectral images via CNNs.

The workflow is shown in **Figure 6**.

## 3 Results and analyses

### 3.1 Spectral analysis and wavelength selection

**Figure 7:**
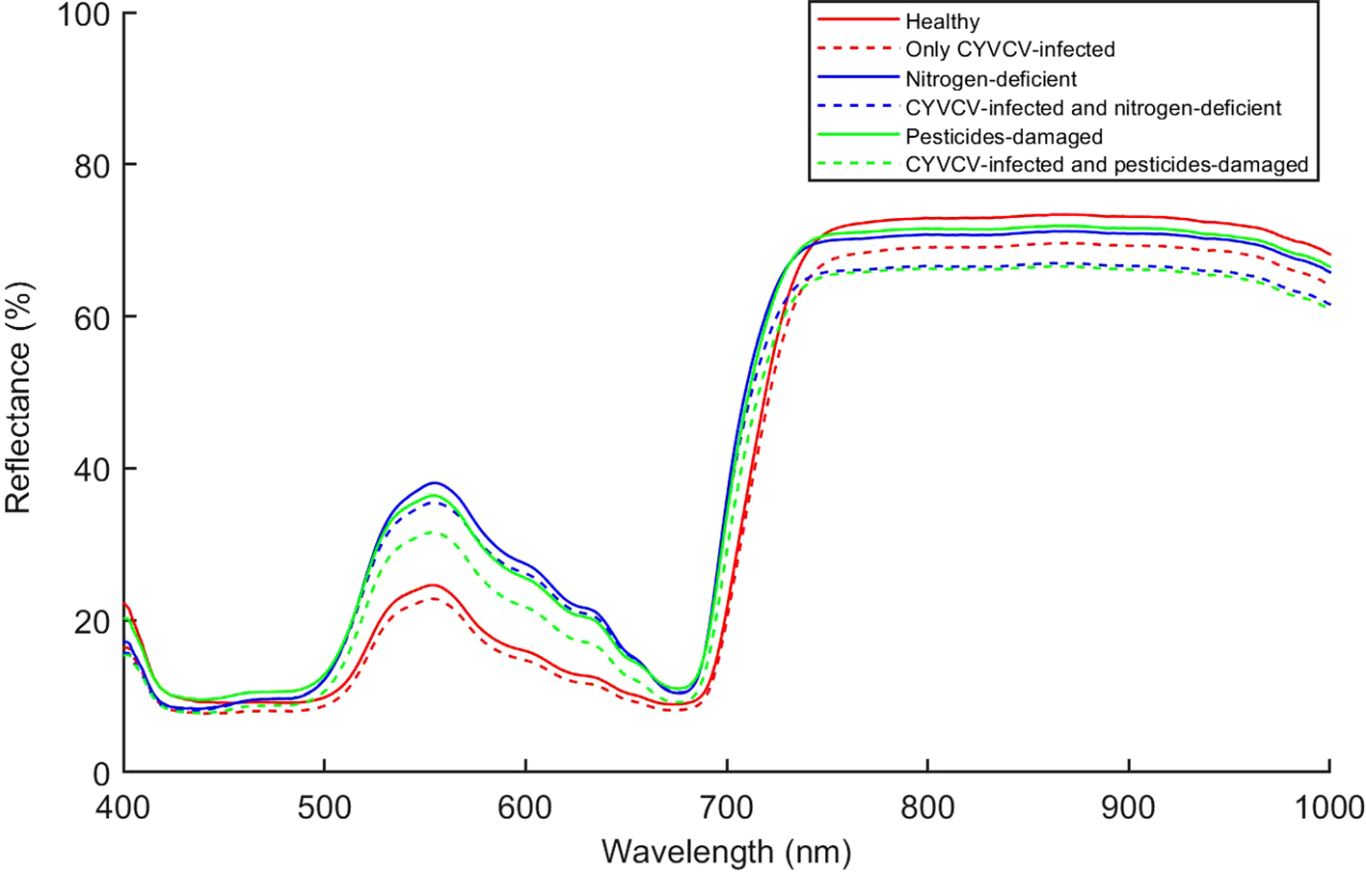
The average lemon-leaf spectra of different types.

A lemon tree can often be infected with multiple diseases at the same time. In this study, we analyzed the types of lemon leaves infected with composite diseases, including YVCD, in field environments. **Figure 7** shows the average spectral reflectance of different types of lemon leaves. The average spectral curves of lemon leaves of different types are generally similar and exhibit the typical spectral reflection characteristics of green plants (Gates et al., 1965; Gitelson and Merzlyak, 1996). Within the 500–680 nm range, we found that the spectral reflectance of healthy leaves and that of those infected only with CYVCV are significantly lower than that of other leaf types. This phenomenon is associated with differences in the chlorophyll content of these leaves (Gitelson et al., 2003) Within the 750–1000 nm range, uninfected leaves show significantly higher reflectance than that of CYVCV-infected leaves, which result from leaf-cell-structure changes and water content caused by CYVCV (PeÑUelas et al., 1993; Wang et al., 2022). Within full bands, the spectral reflectance curves of some leaf types are similar and even overlap at certain wavelengths. These findings indicate differences in terms of the average spectral reflectance among different types of lemon leaves. Therefore, identifying YVCD using leaf spectral information is feasible. However, to achieve higher identification accuracy, it is necessary to further extract and effectively utilize spectral features.

**Figure 8:**
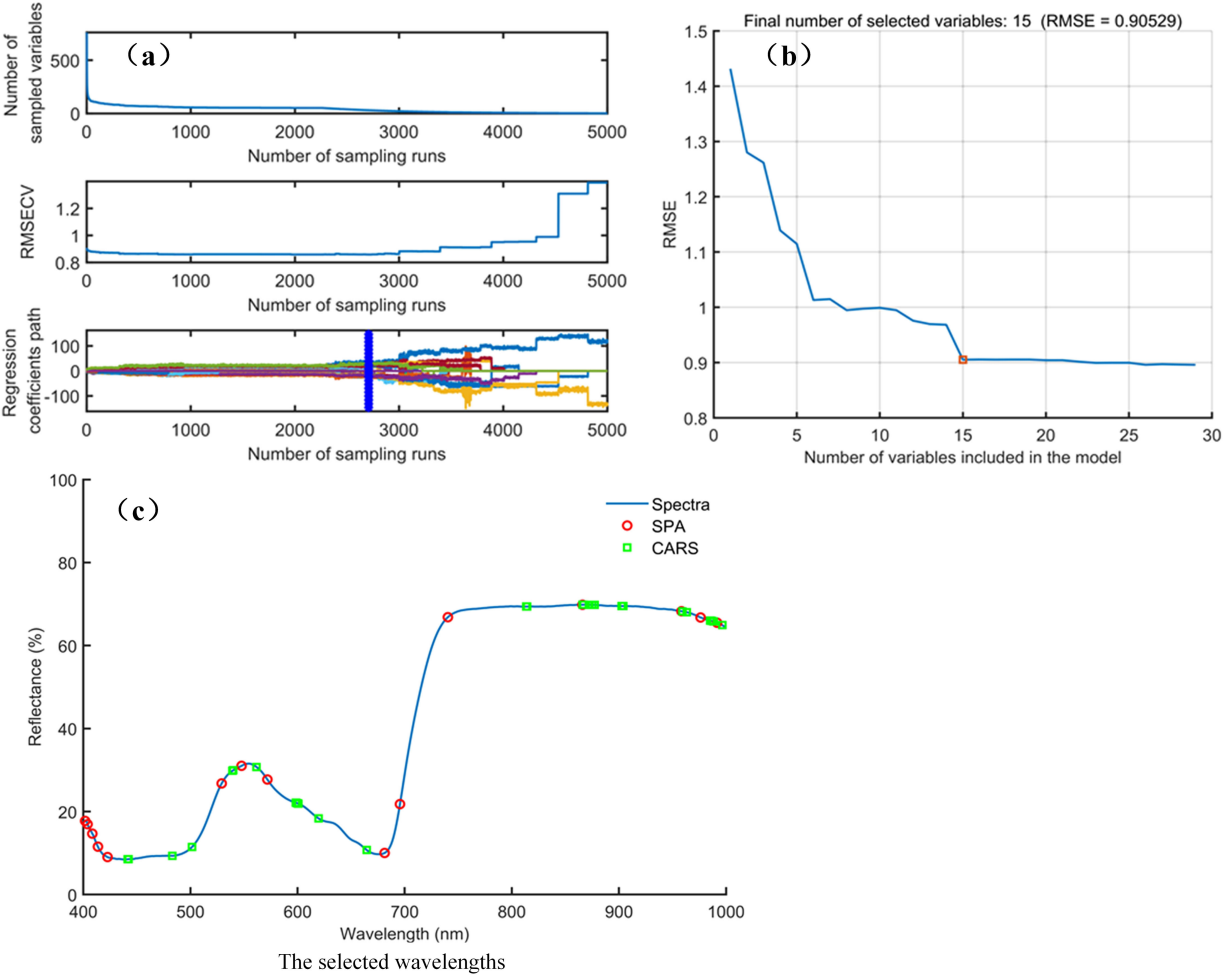
The process and results of feature wavelength extraction. **(a)** The process of CARS; **(b)** The process of SPA; **(c)** The selected wavelengths.

The processes and results of selecting characteristic wavelengths for distinguishing different types of lemon leaves by using the SPA and CARS are shown in **Figure 8**. In process of CARS (**Figure 8a**), as the sampling time increases, the selected wavelength number gradually decreases. The lowest root-mean-square-error-of-cross-validation (RMSECV) value was obtained at the 2708th sampling. CARS selected 30 characteristic wavelengths (441, 442, 483, 501, 539, 540, 562, 598, 599, 600, 601, 620, 665, 813, 814, 866, 867, 872, 875, 877, 902, 904, 959, 963, 985, 986, 987, 988, 996, and 1001 nm), primarily distributed in the 441–665 nm and 813–1001 nm ranges (**Figure 8c**). In process of SPA (**Figure 8b**), it is evident that as the number of selected variables increases, the root mean square error of prediction (RMSEP) decreases. When 15 wavelengths were chosen, the RMSEP showed no significant decrease. The SPA selected 15 characteristic wavelengths (402, 404, 408, 414, 423, 529, 548, 572, 681, 696, 740, 866, 958, 976, and 992 nm), distributed in the “green peak” (550 nm), “red edge,” (680–750 nm) and “high reflection platform” (750–1000 nm) (**Figure 8c**). The results indicate that due to the different characteristic wavelength selection algorithms used, there were inconsistencies in the numbers and positions of the characteristic wavelengths for identifying various types of lemon leaves.

### 3.2 Results of SVM and PLS-DA

**Figure 9:**
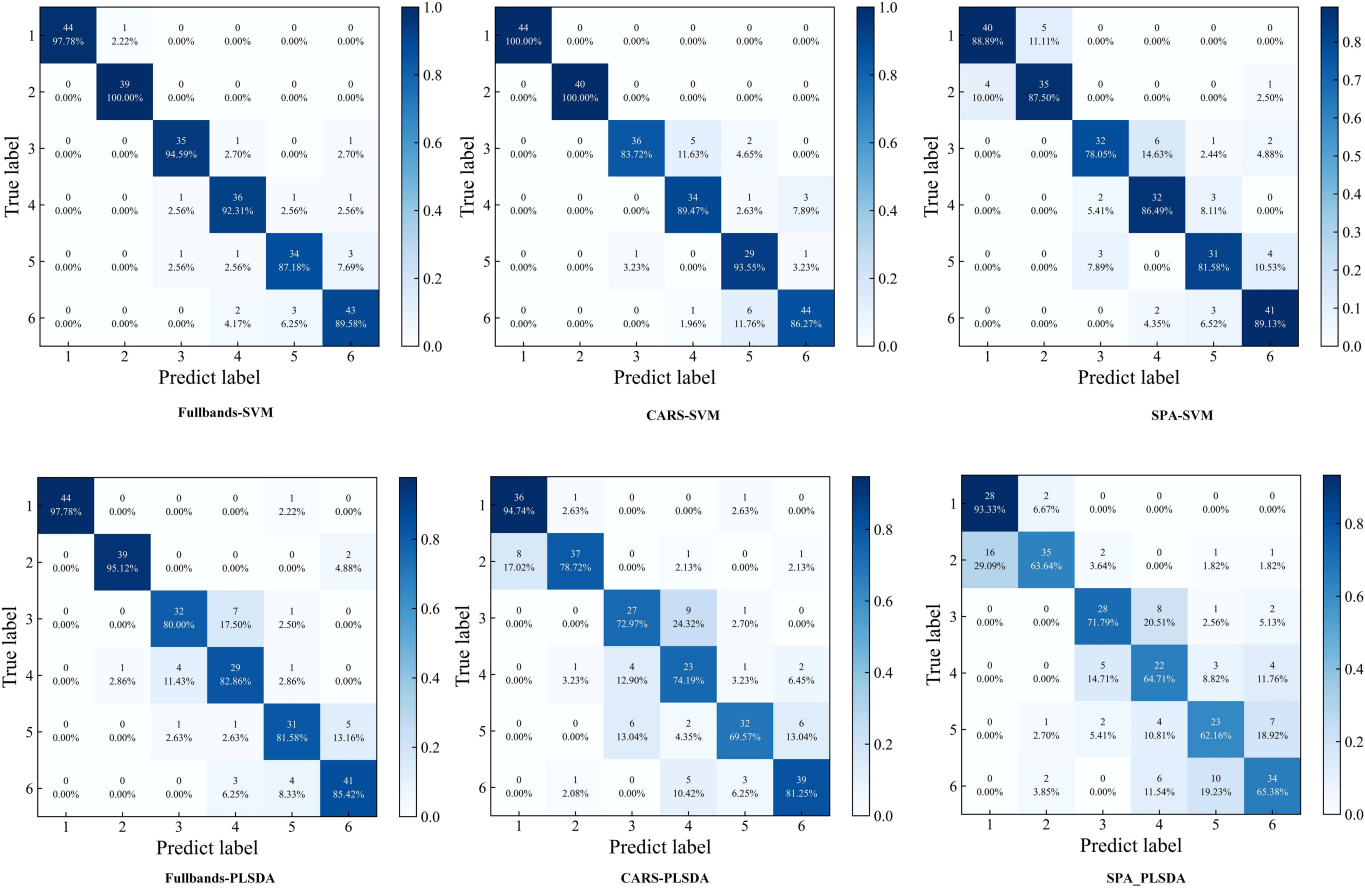
Confusion matrixes of SVM and PLS-DA models. (1:healthy; 2:CYVCV-infected only; 3: nitrogen-deficient; 4: nitrogen-deficient + CYVCV-infected; 5:pesticide-damaged; 6: pesticide-damaged + CYVCV-infected).

The prediction results of SVM and PLS-DA for identifying YVCD by using full-wavelength and characteristic wavelength spectral information are shown in **Table 1** and **Figure 9**. The SVM and PLS-DA models using the full-band spectral information of 761 wavelengths achieved better prediction results, with accuracies of 93.52% and 87.45%, respectively, and exhibited high precision (93.57% and 87.13%), recall (93.53% and 87.25%), and F1-scores (93.54% and 87.10%), indicating balanced classification performance. The SPA-SVM and SPA-PLS-DA models built using the spectral data at the 15 characteristic wavelengths extracted by the SPA achieved the worst prediction results, having accuracies of only 85.43% and 69.83%, respectively. Their precision (85.27% and 70.17%), recall (85.32% and 68.86%), and F1-scores (85.23% and 68.64%) also demonstrated notable declines, suggesting poor generalization ability for these simplified models. Extraction of characteristic wavelengths by CARS and the SPA did not improve the identification performance. This may be the result of selecting only part of the wavelengths, with some spectral information relevant to disease identification potentially lost, thus leading to a decrease in model performance (Qiao et al., 2022). The CARS-SVM model built with the 30 wavelengths selected by CARS achieved a prediction accuracy of 91.90%, which is relatively close to the full-band SVM prediction accuracy. CARS selected 30 variables from 761 wavelengths, greatly reducing the complexity of inputs and computations, which is significant for reducing computational costs in practical applications. Therefore, the CARS-SVM model is the optimal spectral-data model.

**Table 1:** The prediction results of SVM and PLS-DA models.

Models	Input	Numbers of input	Accuracy (%)	Precision (%)	Recall (%)	F1 (%)
SVM	Full bands	761	**93.52**	**93.57**	**93.53**	**93.54**
	CARS	30	91.90	92.17	91.71	91.69
	SPA	15	85.43	85.27	85.32	85.23
PLS-DA	Full bands	30	87.45	87.13	87.25	87.10
	CARS	10	78.54	78.57	78.38	78.01
	SPA	11	68.83	70.17	68.86	68.64

Bold entries highlight the Full bands-SVM model’s overall best performance across key metrics (accuracy, precision, recall, F1-score) among all compared models.

The confusion matrix showed that the models based on full-bands and CARS achieved the highest accuracy in identifying healthy and only CYVCV-infected leaves in the test set. All models exhibited misclassifications among nitrogen-deficient, nitrogen-deficient + CYVCV-infected, pesticide-damaged, and pesticide-damaged + CYVCV-infected leaves. This misclassification is linked to the chlorophyll content of the samples. Studies have demonstrated that the chlorophyll content of healthy and that of only CYVCV-infected leaves are significantly higher than that of other categories, whereas the difference in chlorophyll content among other categories is less pronounced (Wang et al., 2022; Li et al., 2022). As shown by the spectral curves in **Figure 7**, the spectral reflectance of healthy and only CYVCV-infected leaves within the 400–700 nm wavelength range—closely associated with chlorophyll content—is significantly lower than that of other leaves. In contrast, the differences in spectral reflectance between the other types were not significant within 400–700 nm range. In SPA-SVM, healthy leaves were often confused with only CYVCV-infected leaves. This is attributed to the minimal difference in the chlorophyll content of these two types. Furthermore, the SPA only selected four wavelengths (866, 958, 976, and 992 nm) within the 800–1000 nm range, which is related to the leaf tissue structure. These four wavelengths may be insufficient to fully distinguish between all healthy and only CYVCV-infected leaves. These results suggest that spectral information-based classification models can achieve high accuracy in identifying plant leaves with significantly reduced chlorophyll contents, but are less effective in distinguishing complex diseases.

### 3.3 Results of CNNs

**Figure 10:**
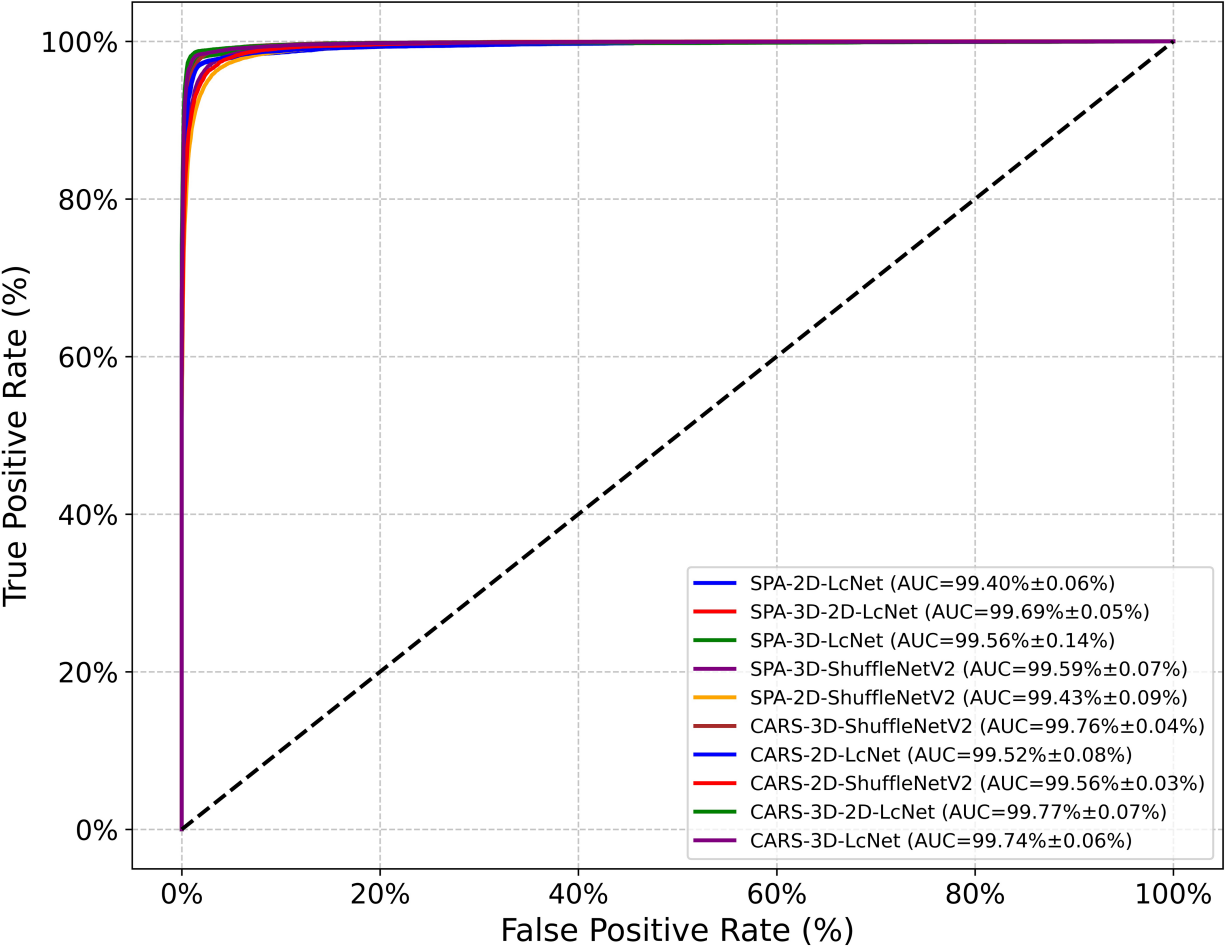
ROC curves of CNN models.

In this study, hyperspectral images composed of gray images at SPA and CARS feature wavelengths were used as the dataset to train CNN models. As shown in **Tables 2**, **3**, and **Figure 10**, the performance evaluation metrics of the CNN models in testing set are presented. The mean accuracy, precision, recall, F1 score and MCC values of all CNN-based models exceeded 91.72%, with the 3D-2D-LcNet model demonstrating the highest overall performance. Specifically, for CARS input, 3D-2D-LcNet achieved 97.35 ± 0.21% accuracy, 97.48 ± 0.22% precision, 97.36 ± 0.20% recall, 97.37 ± 0.21% F1-score, and 96.84 ± 0.25% MCC. These values significantly surpass both 2D counterparts (2D-LcNet: 95.97 ± 0.15% accuracy; 2D-ShuffleNetV2: 93.90 ± 0.42% accuracy) and other 3D models (3D-ShuffleNetV2:95.91 ± 0.20% accuracy). For SPA input, the 3D-2D-LcNet outperformed all 2D and most 3D architectures across metrics, with 96.86 ± 0.06% accuracy, 96.99 ± 0.08% precision, 96.89 ± 0.06% recall, 96.90 ± 0.06% F1-score, and 96.14 ± 0.15% MCC. The performance differences among the models are also evident in their ROC curves (**Figure 10**). The area under the curve (AUC) is a key metric for evaluating the performance of binary classification models based on ROC curves. As shown in the ROC curves of CNN models, the 3D-2D-LcNet models demonstrated remarkable superiority in classification performance. Specifically, the CARS-3D-2D-LcNet model achieved the highest AUC of 99.77 ± 0.07%, significantly outperforming its 2D counterpart (CARS-2D-LcNet, 99.52 ± 0.08%) and other 3D models such as CARS-3D-ShuffleNetV2 (99.76 ± 0.04%). The SPA-3D-2D-LcNet model also excelled with an AUC of 99.69 ± 0.05%, surpassing SPA-2D models (e.g., SPA-2D-ShuffleNetV2, 99.43 ± 0.09%) and highlighting the robustness of the 3D-2D-LcNet architecture across different input modalities. This performance advantage is attributed to the model’s ability to integrate 3D convolution for capturing spatial-temporal features, enhancing discriminative power between positive and negative classes compared to traditional 2D models. Collectively, the ROC curve analysis further validates that the 3D-2D-LcNet model outperforms other architectures, consistent with the superior metrics observed in accuracy, precision, F1-score and MCC analyses. These results demonstrate that the hybrid 3D-2D-LcNet effectively extract both spectral and spatial features from hyperspectral images, thereby leveraging the advantages of hyperspectral imaging more effectively.

**Table 2:** Accuracy, precision, recall, F1 score and MCC achieved of CNN models.

Models	Input	Accuracy (%)	Precision (%)	Recall (%)	F1 (%)	MCC (%)
3D-2D-LcNet	CARS	**97.35 ± 0.21^a^**	**97.48 ± 0.22 ^a^**	**97.36 ± 0.20 ^a^**	**97.37 ± 0.21^a^**	**96.84 ± 0.25^a^**
	SPA	**96.86 ± 0.06 ^ab^**	**96.99 ± 0.08 ^ab^**	**96.89 ± 0.06 ^ab^**	**96.90 ± 0.06^ab^**	**96.14 ± 0.15^ab^**
3D-LcNet	CARS	97.14 ± 0.21 ^a^	97.27 ± 0.20 ^a^	97.15 ± 0.21^a^	97.16 ± 0.2 ^ab^	96.59 ± 0.26^a^
	SPA	96.65 ± 0.23^abc^	96.77 ± 0.21^abc^	96.68 ± 0.23^ab^	96.67 ± 0.23^abc^	96.10 ± 0.31^ab^
3D-ShuffleNetV2	CARS	95.91 ± 0.2^abc^	96.03 ± 0.23^abc^	95.93 ± 0.21^abc^	95.94 ± 0.21^abc^	95.10 ± 0.26^abc^
	SPA	94.58 ± 0.34^bc^	94.68 ± 0.3^bc^	94.66 ± 0.34^bc^	94.63 ± 0.34^bc^	93.50 ± 0.40^bc^
2D-LcNet	CARS	95.97 ± 0.15^abc^	96.11 ± 0.15^abc^	96.00 ± 0.14^abc^	95.99 ± 0.14^abc^	95.19 ± 0.18^abc^
	SPA	95.81 ± 0.31^abc^	95.92 ± 0.29^abc^	95.85 ± 0.31^abc^	95.84 ± 0.31^abc^	94.99 ± 0.36^abc^
2D-ShuffleNetV2	CARS	93.90 ± 0.42^c^	94.04 ± 0.44^c^	93.97 ± 0.40^bc^	93.96 ± 0.4^c^	92.70 ± 0.50^bc^
	SPA	93.09 ± 0.54^c^	93.19 ± 0.53^c^	93.19 ± 0.5^c^	93.15 ± 0.53^c^	91.72 ± 0.65^c^

Superscript letters indicate statistically significant differences between models (p< 0.05); MCC, Matthews Correlation Coefficient. Bold entries highlight the 3D-2D-LcNet model’s overall best performance across key metrics (accuracy, precision, recall, F1-score, MCC) among models with the same input.

**Table 3:** Train time, test time, param and FLOPs of CNN models.

Models	Input	Train time (s)	Test time (s)	Params (M)	FLOPs (G)
3D-2D-LcNet	CARS	1716.62 ± 119.85^ab^	10.46 ± 0.31^de^	0.26	8.42
	SPA	**940.70 ± 23.67^abc^**	**9.23 ± 0.33^f^**	**0.26**	**4.92**
3D-LcNet	CARS	1793.97 ± 148.83^a^	11.07 ± 0.47^d^	0.27	8.91
	SPA	943.66 ± 22.19^abc^	10.04 ± 0.11^ef^	0.27	5.41
3D-ShuffleNetV2	CARS	1831.15 ± 104.35^a^	19.66 ± 0.67^a^	0.22	8.94
	SPA	1052.1 ± 20.01^abc^	18.75 ± 0.98^ab^	0.22	4.59
2D-LcNet	CARS	1688.02 ± 160.65^abc^	10.21 ± 0.24^def^	0.25	2.22
	SPA	889.03 ± 43.89^c^	9.30 ± 0.25^f^	0.25	1.79
2D-ShuffleNetV2	CARS	1716.48 ± 33.6^abc^	18.12 ± 0.57^bc^	0.21	2.54
	SPA	916.64 ± 16.3^bc^	17.26 ± 0.27^c^	0.21	1.89

Superscript letters indicate statistically significant differences between models (p< 0.05); Param (M), Number of parameters in millions; FLOPs (G), Floating point operations in giga. Bold entries highlight the SPA-3D-2D-LcNet model’s overall best performance across efficiency metrics (Train/Test time, Params, FFLOPs) among all compared models.

In terms of model complexity, the parameter counts of the 3D convolution models (3D-2D-LcNet, 3D-LcNet, and 3D-ShuffleNetV2) were not significantly higher than those of their 2D counterparts. For instance, the 3D-2D-LcNet and 3D-LcNet both have 0.26 million and 0.27 million parameters, respectively, which is comparable to the 0.25 million parameters of 2D-LcNet. Regarding training cost, the 3D convolution models (3D-LcNet and 3D-ShuffleNetV2) exhibited higher FLOPs, training time, and testing time than 2D models (2D-LcNet and 2D-ShuffleNetV2). Specifically, the 3D-LcNet required 1,793.97 seconds of training time on the CARS dataset, which is 6.2% longer than the CARS-2D-LcNet (1,688.02 seconds). However, the hybrid 3D-2D-LcNet demonstrated a unique advantage in that its training time and test time on both CARS and SPA datasets were shorter than that of pure 3D models. The hybrid 3D-2D-LcNet mitigated this trade-off by reducing FLOPs by 5.5% (8.42G vs. 8.91G) compared to 3D-LcNet while maintaining comparable accuracy.

The different dimension sizes in hyperspectral images result in differences in terms of the computational load and recognition accuracy. In this study, we compared the effects of hyperspectral image datasets at different characteristic wavelengths on CNN model training and performance. In terms of complexity, CNN models based on CARS clearly required significantly more computational resources than those based on SPA—particularly for CNNs incorporating 3D convolution modules. Regarding predictive performance, the evaluation metrics revealed no significant differences between the models with the same CNN architecture but different training datasets. These results demonstrate that hybrid 3D-2D-LcNet effectively balances computational complexity with feature extraction efficacy. Its robustness to spectral data of different wavelengths and efficient inference capabilities make it a versatile solution for hyperspectral images analysis.

**Figure 11:**
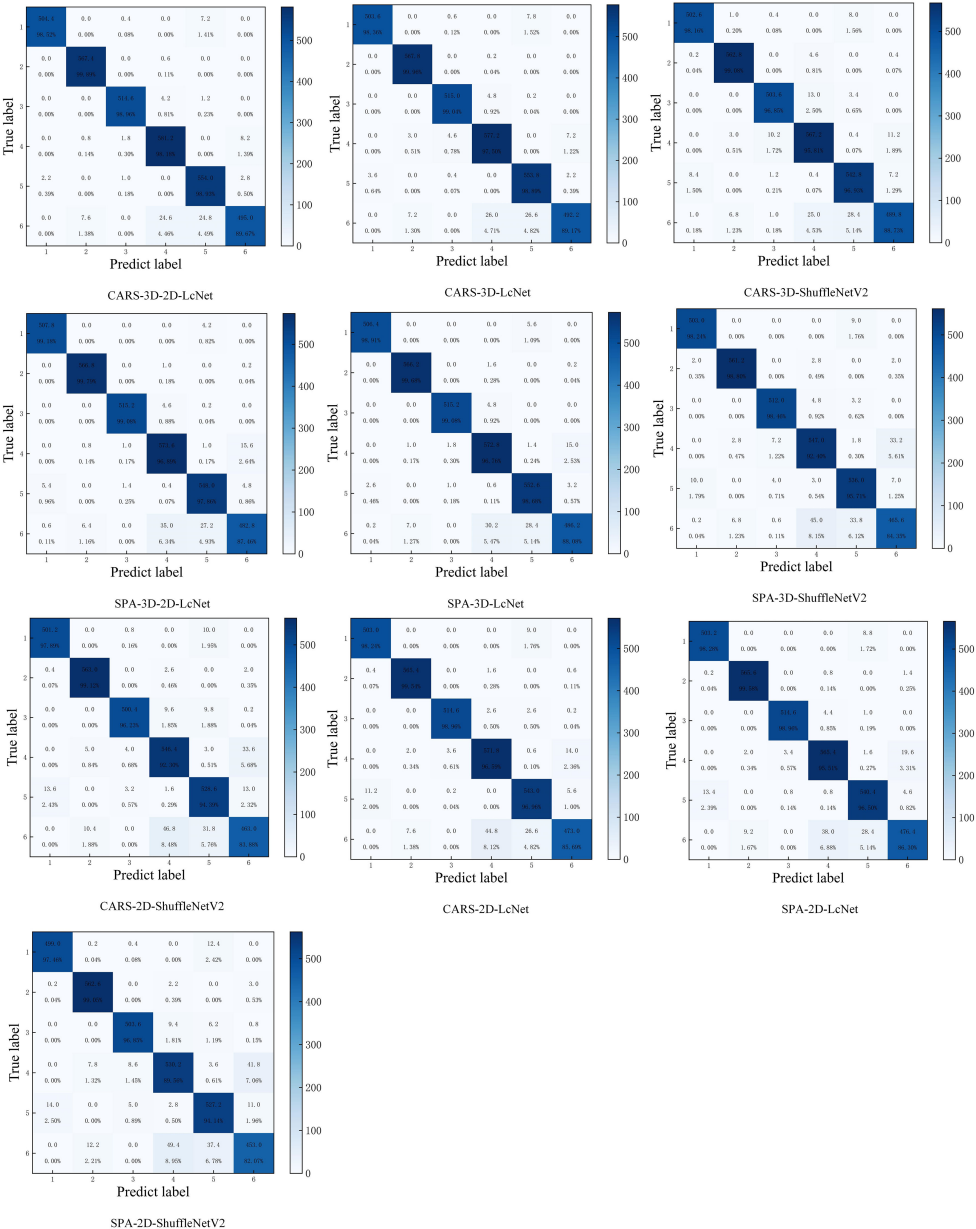
Confusion matrix of CNN models. (The confusion matrix is generated by averaging the results of five repeated. (1: healthy; 2: CYVCV-infected only; 3: nitrogen-deficient; 4: nitrogen-deficient + CYVCV-infected; 5: pesticide-damaged; 6: pesticide-damaged + CYVCV-infected).

The confusion matrix (**Figure 11**) shows that all the models achieved classification accuracies ranging from 93.07% to 100% of the five sample categories: healthy, only CYVCV-infected, nitrogen-deficient, nitrogen-deficient+ CYVCV-infected, and pesticide-damaged. However, the classification accuracy of pesticide-damaged+CYVCV-infected samples ranged from 77.17% to 89.86%, with misclassifications into only diseased, nitrogen-deficient+ CYVCV-infected, and pesticide-damaged categories. Overall, both traditional hyperspectral image classification methods and CNNs showed low accuracy in identifying pesticide-damaged+CYVCV-infected samples, and these samples were easily confused with only diseased, nitrogen-deficient+CYVCV-infected, and pesticide-damaged samples. This may be due to overlapping spectral features and insufficient sensitivity to subtle biochemical and structural variations. When the pesticide-damaged characteristics of a sample are not obvious, it is misclassified as CYVCV-infected, specifically as only diseased or nitrogen-deficient + CYVCV-infected. When the CYVCV-infected characteristics are not obvious, it was misclassified as pesticide-damaged. Only when both the pesticide-damaged and CYVCV-infected characteristics were prominent is it correctly classified as pesticide-damaged+CYVCV-infected.

In summary, among all CNN models, the CARS-3D-2D-LcNet has consistently achieved the highest accuracy of 97.35 ± 0.21%, with no statistically significant difference from the SPA-3D-2D-LcNet. In contrast, the SPA-3D-2D-LcNet achieved an optimal balance between accuracy and efficiency by reducing computational resource consumption by 41.6% (4.92G vs. 8.42G FLOPs) and accelerating inference speed by 11.8%. Acquiring and transmitting high-dimensional hyperspectral images in practical deployment imposes higher demands on device storage, transmission, and computational capabilities. In resource-constrained scenarios, the SPA-3D-2D-LcNet significantly reduces deployment complexity and costs while maintaining nearly equivalent accuracy. Although the CARS-3D-2D-LcNet demonstrated peak performance under laboratory conditions, the SPA-3D-2D-LcNet better aligns with real-world requirements for limited resources and high real-time demands, offering a triple optimal solution in terms of accuracy, efficiency, and cost for practical implementation.

### 3.4 Comparison between deep learning and machine learning

Traditional hyperspectral image classification methods that use spectral information and machine learning are still widely utilized (Abdulridha et al., 2019; Qiao et al., 2022). Our results indicated a notable performance gap between CNN-based models and traditional machine learning models. The CNN-based models demonstrated accuracies ranging from 93.90% to 97.35%. Among CNN-based models, the CARS-3D-2D-LcNet model showed high accuracy across various classes, with most samples in each class being correctly classified. It achieved accuracies above 98.13% for healthy, only CYVCV-infected, nitrogen-deficient, nitrogen-deficient+CYVCV-infected, and pesticide-damaged samples, and maintaining 89.67% accuracy for pesticide-damaged+ CYVCV-infected samples. In contrast, the accuracies of machine learning-based models spanned from 68.83% to 93.52%. Among machine learning-based models, the fullbands-SVM outperformed best, while it achieved 87.18-97.78% accuracies for healthy, only CYVCV-infected, nitrogen-deficient, nitrogen-deficient + CYVCV-infected, and pesticide-damaged samples, only 85.29% of pesticide-damaged samples were correctly classified. This highlights that CNNs, with their ability to automatically extract hierarchical spatial-spectral features, outperform traditional methods in classifying complex composite diseases based on hyperspectral images. Meanwhile, traditional methods, which rely mainly on hand-engineered spectral features, can still deliver acceptable results for single-disease identification.

Although these CNN models have achieved good performance in the identification of YVCD based on hyperspectral images, hyperspectral data of high dimensionality requires substantial computational power for training. In particular, in 3D-CNN, the computational complexity increases exponentially. The high memory consumption and slow computational speed of CNN models during training and inference also make it difficult to deploy them on portable devices or edge devices (such as field monitoring devices). The proposed 3D-2D-LcNet, which combines lightweight modules and hybrid 3D-2D convolutions, alleviates the computational burden of traditional 3D-CNNs to some extent. However, the computational cost of 3D-2D-LcNet is still relatively high, and there is still a gap before it can be deployed on-site. In contrast, machine learning models exhibit unique advantages in field application due to their low computational complexity, low memory usage, and fast computation speed. They do not require large amounts of labeled data and can complete classification through artificially designed spectral features (such as spectral indices and band combinations), making them suitable for rapid deployment in resource-limited environments. This might account for why traditional hyperspectral image classification methods are still widely employed. However, the traditional methods fail to fully explore the subtle spectral-spatial information associations in hyperspectral data and cannot achieve satisfactory classification accuracy for complex composite diseases. Moreover, the performance of machine learning models can drop significantly when spectral data distribution shifts due to changes in environmental conditions. In contrast, although CNNs are limited in computational resources, their end-to-end learning mechanism can automatically adapt to data changes, giving them unique competitiveness in disease identification based on hyperspectral images.

## 4 Conclusion

In this study, we explored efficient and accurate methods for identifying YVCD in lemons using hyperspectral images. The results indicate that the four CNN architectures used in this study significantly outperformed traditional machine learning methods for YVCD identification. Among them, the innovative 3D-2D-LcNet stands out. By effectively integrating spatial and spectral feature extraction, it optimally balances computational complexity and feature extraction efficiency, significantly reducing computational load while maintaining high accuracy in extracting both spectral and spatial features from hyperspectral images. The CARS-3D-2D-LcNet model demonstrated best performance in identifying YVCD but faced challenges in practical deployment due to substantial computational demands and high-dimensional data requirements. In contrast, the SPA-3D-2D-LcNet achieves comparable performance with 41.6% lower FLOPs and 50% reduced data dimensionality, bringing hope for the field application of this hyperspectral image-based disease detection method. In conclusion, leveraging advanced CNN architectures can enhance the performance of hyperspectral imaging-based disease detection models. The 3D-2D-LcNet offers a highly effective and accurate approach for identification of YVCD in lemons, demonstrating its potential for practical applications in agricultural disease detection.

## Data Availability

The raw data supporting the conclusions of this article will be made available by the authors, without undue reservation.
